# Coronary microvascular function in male physicians with burnout and job stress: an observational study

**DOI:** 10.1186/s12916-023-03192-z

**Published:** 2023-12-01

**Authors:** Roland von Känel, Mary Princip, Sarah A. Holzgang, Chrysoula Garefa, Alexia Rossi, Dominik C. Benz, Andreas A. Giannopoulos, Philipp A. Kaufmann, Ronny R. Buechel, Claudia Zuccarella-Hackl, Aju P. Pazhenkottil

**Affiliations:** 1 Department of Consultation-Liaison Psychiatry and Psychosomatic Medicine, University Hospital Zurich, University of Zurich, Culmannstrasse 8, CH-8091 Zurich, Switzerland; 2 Cardiac Imaging, Department of Nuclear Medicine, University Hospital Zurich, University of Zurich, Zurich, Switzerland; 3 Department of Cardiology, University Hospital Zurich, University of Zurich, Zurich, Switzerland

**Keywords:** Burnout, Cardiovascular disease, Job stress, Myocardial blood flow, Physicians, Psychological stress

## Abstract

**Background:**

As a professional group, physicians are at increased risk of burnout and job stress, both of which are associated with an increased risk of coronary heart disease that is at least as high as that of other professionals. This study aimed to examine the association of burnout and job stress with coronary microvascular function, a predictor of major adverse cardiovascular events.

**Methods:**

Thirty male physicians with clinical burnout and 30 controls without burnout were included. Burnout was assessed with the Maslach Burnout Inventory and job stress with the effort-reward imbalance and overcommitment questionnaire. All participants underwent myocardial perfusion positron emission tomography to quantify endothelium-dependent (cold pressor test) and endothelium-independent (adenosine challenge) coronary microvascular function. Burnout and job stress were regressed on coronary flow reserve (primary outcome) and two additional measures of coronary microvascular function in the same model while adjusting for age and body mass index.

**Results:**

Burnout and job stress were significantly and independently associated with endothelium-dependent microvascular function. Burnout was positively associated with coronary flow reserve, myocardial blood flow response, and hyperemic myocardial blood flow (*r* partial = 0.28 to 0.35; *p*-value = 0.008 to 0.035). Effort-reward ratio (*r* partial =  − 0.32 to − 0.38; *p*-value = 0.004 to 0.015) and overcommitment (*r* partial =  − 0.30 to − 0.37; *p*-value = 0.005 to 0.022) showed inverse associations with these measures.

**Conclusions:**

In male physicians, burnout and high job stress showed opposite associations with coronary microvascular endothelial function. Longitudinal studies are needed to show potential clinical implications and temporal relationships between work-related variables and coronary microvascular function. Future studies should include burnout and job stress for a more nuanced understanding of their potential role in cardiovascular health.

## Background

Occupational burnout can be described as a condition characterized by a sense of exhaustion, detachment from one’s work, and decreased productivity resulting from prolonged exposure to unmanaged job stress [1]. This conceptual framing of burnout is bolstered, for instance, by a recent systematic review of longitudinal studies, which demonstrates that high job demands and negative job attitudes are predictors of occupational burnout with effect sizes ranging from small to medium [2]. With a prevalence of about 50%, burnout is high in physicians [3] who also report higher job stress compared with the general population [4, 5]. Physician burnout is associated with poor mental health, including depression and suicidal ideation, reduced productivity, early job retirement, and lower patient satisfaction [6]. Similar negative effects on physician well-being and patient care have been found to result from increased job stress [7, 8]. Accumulating evidence also shows associations of both burnout and job stress with an increased risk of somatic diseases [9, 10], particularly coronary heart disease (CHD) [11, 12]. Specifically, among 8838 seemingly healthy employees in Israel, elevated initial levels of burnout were indicative of an elevated CHD risk after a follow-up of 3.4 years [11]. This association remained significant even after adjusting for demographics, cardiovascular risk factors, job stress, and depression [11]. Despite their medical background, physicians have at least as high a risk of cardiovascular disease (CVD) as other professionals [13, 14].

Explanations for the observed associations of burnout and job stress with an increased CHD may include unhealthy lifestyle behaviors and psychobiological changes like autonomic and neuroendocrine dysfunction and low-grade inflammation [15, 16]. However, the underlying pathophysiology of CHD risk in physicians with burnout and job stress is unknown. Moreover, examining the independent associations of these related constructs may lead to a more nuanced understanding of their impact on cardiovascular health. For instance, one meta-analysis found that the cortisol awakening response was negatively associated with burnout, but positively so with job stress [17].

With respect to cardiovascular health, the coronary microcirculation plays a pivotal role in modulating coronary blood flow to meet the oxygen demands of the heart [18]. Coronary microvascular dysfunction is expressed as reduced coronary flow reserve (CFR) which can be non-invasively assessed through positron emission tomography (PET) myocardial perfusion imaging [19]. Reduced CFR serves as an indicator of coronary artery disease severity and has been prospectively associated with an increased risk of major adverse cardiovascular events and all-cause mortality [20]. To the best of our knowledge, there have been no studies that have explored the correlation between both burnout and job stress with CFR. Therefore, the aim of this study was to examine the independent association of burnout and job stress with CFR in male physicians.

## Methods

### Participants

The local ethics committee Zurich (BASEC-Nr. 2018–01974) approved the study, and written informed consent was obtained from all participants. Between 09/2019 and 12/2021, we recruited male physicians in Switzerland to investigate the relation of burnout to cardiovascular health. Table 1 provides names and explains in straightforward language key parameters that were measured in this study. Various channels, including hospitals, clinics, physician associations, and direct email communication, were used to reach potential participants. Interested physicians were provided with study details and objectives through text/flyer and had the option to contact the study management. The study aimed to enroll 60 participants who were assigned to either the burnout group or the healthy control group, with 30 participants in each group. The sample size was determined based on a previous study from our institution, showing that *n* = 23 patients with obstructive sleep apnea in each of two groups were required to detect a group difference (*p* < 0.05) in adenosine-induced hyperemic MBF of clinical significance (0.75 ± 0.85 mL/min/g) [21]. To our knowledge, there is no data available in the literature to perform a formal power analysis regarding CFR, which was the primary outcome variable of this study.

**Table 1:** Key parameter explanations

Coronary microvascular function	Coronary microvascular function refers to the health of the small blood vessels in the heart that supply blood to the heart muscle. To assess this, doctors use imaging of the heart together with for example application of adenosine, a pharmacological substance to dilate the blood vessels, or the cold pressor test (see below). These tests can be used to assess how well the heart’s small blood vessels respond to stress and ensure proper blood flow to the heart muscle (i.e., myocardial blood flow, abbreviated as MBF), which provides information about cardiovascular health
Coronary flow reserve	An index of how well the coronary arteries and small blood vessels can adjust and increase blood flow during stress compared to the resting blood flow. It is calculated by dividing the peak stress MBF by the resting MBF. A higher ratio is generally considered beneficial since it indicates that the tiny heart vessels can dilate and allow more blood to flow to the heart muscle when needed
Myocardial blood flow response	The change (Δ sign for change) from MBF at rest to the peak stress MBF response. ΔMBF is calculated by subtracting resting MBF from the peak stress MBF response
Hyperemic myocardial blood flow	The peak volume of blood that the heart muscle can receive during the maximum stress-induced dilation of tiny heart vessels
Cold pressor test	In the cold pressor test, a part of the body (e.g., the arm or foot) is immersed in ice-cold water for a few minutes to see how the cardiovascular system reacts to the stress of the cold, which activates the sympathetic nervous system
Occupational burnout	Occupational burnout is a condition characterized by extreme fatigue, mental distance from work, and a sense of diminished achievement or productivity on the job. This state makes individuals feel both physically and mentally exhausted while experiencing a decline in job satisfaction and accomplishment
Effort-reward ratio	This measure for job stress compares the effort a person puts into their work (e.g., intensity and time spent on tasks) with the reward they receive for it (e.g., recognition, salary). A higher ratio indicates more stress in the workplace and is used in occupational health research to assess the impact of job stress on mental and physical health
Overcommitment	A pattern of behavior in which people tend to exceed their available resources by taking on more work or responsibility at work than they can effectively manage. This leads to increased job stress and potentially negative health consequences

Participants were screened and assigned to the burnout or healthy control group using the MBI-Human Services Survey (MBI-HSS) [22] and the Patient Health Questionnaire (PHQ)-9 [23] via telephone interview, with additional inclusion and exclusion criteria reviewed. To determine the cutoff points for group assignment, we referred to a previous systematic review on physician burnout [2]. For the clinical burnout group, we used emotional exhaustion (EE) ≥ 27 and/or depersonalization (DP) ≥ 10 (with a minimum EE score ≥ 20) as cutoff points. For the control group, the cutoff points were EE < 16 and DP < 7. The personal accomplishment (PA) subscale was not used for group assignment as it develops largely independently of EE and DP [24]. In addition, for the burnout group, a PHQ-9 score of ≤ 14 was required, indicating at most moderate depressive symptoms, experienced stress at work, and undue exhaustion for at least 6 months prior to enrollment in the study [25]. For the control group, a PHQ-9 score of ≤ 10 was required, indicating at most mild depressive symptoms [23]. We sought to match controls with participants with burnout in terms of age in the range of 5 years, body mass index (BMI) in the range of 5 kg/m^2^, and family history of early CVD in first-degree relatives (< 55 years in men, < 65 years in women).

Both groups had additional inclusion criteria, which required non-smoking for at least 5 years and age between 28 and 65 years, as in Switzerland most physicians do not practice medicine for at least 6 months before age 28, and 65 is the official retirement age. Exclusion criteria for both groups included a previous episode of clinical depression or burnout; known heart disease; familial hypercholesterolemia; type I or type II diabetes; stage II hypertension; renal insufficiency; any known active serious disease; intake of lipid-lowering, antihypertensive, or antidiabetic drugs; BMI ≥ 35 kg/m^2^; chronic risky alcohol consumption (≥ 4 standard drinks per day); allergy to iodine-containing contrast media; contraindications for adenosine, beta-blockers, or nitrates; a medication that interferes with blood biomarker levels (e.g., corticosteroids, anticoagulants); and the decision to waive information about clinically relevant cardiac imaging findings.

### Positron emission tomography for myocardial blood flow assessment

Participants were imaged with the latest generation PET-CT scanner (Discovery MI, GE Healthcare, Waukesha, WI, USA) using ^13^N-ammonia as a flow tracer at the Department of Nuclear Medicine, University Hospital of Zurich. We used adenosine infusion and the cold pressor test (CPT) as pharmacological and sympathetic stressors. MBF was assessed at rest, during adenosine-induced hyperemia (reflecting primarily endothelium-independent vasodilation), and in response to standardized CPT (reflecting primarily endothelium-dependent vasodilation).

Beginning with the intravenous bolus administration of ^13^N-ammonia, serial dynamic and static PET images were acquired for 20 minutes (min); 10 min later, adenosine (at a continuous rate of 140 μg/kg/min) was administered intravenously for 6 min. Three minutes into the adenosine infusion, a second dose of ^13^N-ammonia was injected and images were recorded in the same acquisition sequence. Ten minutes later, a CPT was performed by immersing the participant’s right foot and lower half of the calf in a container filled with ice water (4° C) for 2 min [26]. A third dose of ^13^N-ammonia was administered after 60 s into the CPT, and PET image acquisition was performed. Heart rate, blood pressure, and a 12-lead electrocardiogram were recorded at baseline and throughout the adenosine infusion and CPT. MBF at rest (corrected for rate-pressure product) and during adenosine-stress and CPT was calculated using commercially available software (QPET 2017.7, Cedars-Sinai Medical Center, Los Angeles, CA, USA). Each participant’s global CFR (primary outcome) was calculated as the ratio of stress to rest absolute MBF for the whole left ventricle. As secondary outcomes, we also examined MBF response (i.e., the difference between stress and rest MBF (ΔMBF)) that is not influenced by MBF at rest, and hyperemic MBF (i.e., the maximal flow achieved during hyperemia) [27].

### Psychometric assessment

For the analyses, we used burnout and depressive symptom scores that were obtained on the same day as MBF measurements, because these may be more closely associated with MBF than scores obtained through the previous telephone interview. One participant lacked this information, so we used the symptom scores collected during the telephone screening.

#### Burnout

We used a validated German version of the 22-item MBI-HSS, which includes three subscales assessing EE (nine items), DP (five items), and PA (eight items) [22]. Participants rated each item on a scale ranging from 1 (“never”) to 7 (“daily”). While EE examines the sensation of exhaustion and energy depletion that arise from work, DP assesses a cynical and detached attitude towards work/patients, and PA measures feelings of competence and successful job performance as a physician. In our sample, Cronbach’s *α* was 0.95, 0.87, and 0.77 for the EE, DP, and PA subscales, respectively.

#### Job stress

To assess job stress, we used the validated German short form of the Effort-Reward Imbalance (ERI) and overcommitment (OC) questionnaire, consisting of three items regarding work effort, seven items concerning work-related rewards, and six items pertaining to OC to work [28]. The ERI and OC model posits that job stress with consequences for CVD [6, 7] stems from two factors: a lack of reciprocity in terms of high cost and low gain, reflected by the ER ratio (extrinsic component), and OC to high achievement (intrinsic component) [29]. Moreover, employees who report both a high level of extrinsic and intrinsic job stress are thought to be at the highest risk of CVD [30]. Each questionnaire item is rated on a 4-point Likert scale ranging from 1 (“strongly disagree”) to 4 (“strongly agree”). Higher scores on the effort scale (range 3–12) indicate a greater degree of stress caused by high work effort, while lower scores on the reward scale (range 7–28) reflect more stressful experiences due to inadequate rewards. Higher scores on the OC scale (range 6–24) indicate more stress caused by OC. The ER ratio is calculated using a correction factor that accounts for the unequal number of items of the effort and reward scores. A higher ER ratio indicates greater job stress. In our sample, Cronbach’s *α* was 0.76, 0.77, and 0.81 for the effort, reward, and OC scales, respectively.

#### Depressive symptoms

We used the German version of the PHQ-9 questionnaire to measure depressive symptoms in the previous 2 weeks [31]. The scale comprises nine items that are rated by participants on a 4-point Likert scale between 0 (“not at all”) and 3 (“nearly every day”). Higher total scores (range 0–27) indicate greater severity of depressive symptoms. Cronbach’s *α* for the PHQ-9 total scale was 0.79 in our sample.

### Cardiovascular risk and health behavior assessment

We employed the Systematic Coronary Risk Evaluation (SCORE)2 risk prediction algorithm to estimate the 10-year risk of fatal and non-fatal CVD in individuals younger than 70 years without prior CVD or diabetes [32]. Body mass index (BMI) was calculated by dividing measured weight (in kilograms) by measured height (in meters) squared (kg/m^2^). Physical activity was assessed by asking participants how many times (range 0–7) in an average week they engaged in sports activities that caused them to sweat. Intake of alcohol was determined by the number of standard drinks consumed per week.

### Data analysis

Data were analyzed using IBM SPSS Statistics for Windows, Version 29.0 (Armonk, NY: IBM Corp) with a two-tailed significance level of* p* < 0.05. The expectation–maximization algorithm was used to substitute the few missing items (3.4%) in the ERI and OC questionnaire. Because of the non-normal distribution, all measurements of microvascular function were transformed to statistical normality using a two-step procedure described elsewhere [33], preserving the original series mean and standard deviation. Independent samples *t*-test was used to compare health characteristics between the burnout group and the control group. Pearson correlation analysis was calculated to express zero-order correlations between two variables.

Linear regression analyses were modeled with CRF, ΔMBF, and hyperemic MBF as dependent variables and burnout (yes/no) and job stress as independent variables, adjusting for age and BMI. All independent variables were entered in one block. We included the “constant” (also known as the “intercept”) in all regression models, which represents the expected value of the dependent variable when all independent variables are set to zero. The inclusion of the constant ensures that the model is “unbiased” as the mean of the residuals (i.e., the differences between the observed and predicted values) is exactly zero, thereby contributing to the statistical rigor of the analysis. Adjustment for multiple comparisons was carried out for the multivariable associations of burnout and both job stress measures (ER ratio and OC) with CFR, which was the predefined primary outcome variable of our study. Since we assessed CFR using two different methods, significance was considered if the independent associations of burnout, ER ratio, or OC with endothelium-dependent CFR or endothelium-independent CFR was *p* < 0.025. Given that ER ratio and OC tap into distinct aspects of job stress (i.e., extrinsic vs. intrinsic component), we did not apply further *p*-value adjustment for these two measures. All other associations examined were exploratory and aimed to confirm and identify additional relationships between work-related variables and measures of coronary microvascular function, including ΔMBF and hyperemic MBF, supporting findings for CFR, and generating new hypotheses. For job stress, two models were calculated, one for the ER ratio and one for the OC score. Incorporating these models facilitates an assessment of result robustness across two distinct job stress measures, enhancing the statistical rigor of the analysis. When burnout (yes/no) or the ER ratio showed a significant independent association with a particular measure of microvascular function, additional analyses were performed to examine whether EE, DP, PA, effort, or reward would show separate associations with that same measure.

Two complementary analyses were conducted with interaction terms between burnout and job stress and between the ER ratio and OC, respectively, additionally included in the models. The first analysis examined if the relationship between job stress (either ER ratio or OC) and microvascular function would be moderated by the presence or absence of clinical burnout. The second analysis tested the theoretical assumption that physicians who have both high ER ratio (extrinsic work stress) and OC scores (intrinsic work stress) have a greater reduction in microvascular function.

Due to high multicollinearity between the PHQ-9 score and burnout (by study design), and between age and the SCORE2, depressive symptoms and the SCORE2 were not considered as covariates to prevent inaccurate coefficient estimates. Specifically, the Pearson correlation coefficient yielded a value of 0.711 when examining the relationship between burnout and the PHQ-9 score. Additionally, when included in the multivariable model, the PHQ-9 score exhibited a variance inflation factor (VIF) of 2.761. We adjusted for age instead of SCORE2 because, despite our attempts to match groups on age, the burnout group was significantly younger, whereas both groups had a similar SCORE2 that already accounts for age. All VIFs were below 2.5 in multivariable models, indicating no concern for multicollinearity. For instance, in their respective multivariable models, VIFs were 1.642 for burnout and 1.760 for the ER ratio, and 1.602 for burnout and 1.620 for OC. One case was omitted for all models with the ER ratio because Mahalanobis distance indicated an influential outlier. Effect sizes of associations are expressed as correlation coefficients, where *r* = 0.1, 0.3, and 0.5 reflect small, medium, and large effects, respectively.

## Results

### Participant characteristics

Table 2 shows that physicians with burnout were significantly younger than their counterparts without burnout (*p* = 0.012), whereas the other cardiovascular risk factors studied did not show a significant group difference (*p*-values > 0.05). In contrast, psychometric scores differed significantly between the two groups. Physicians with burnout reported higher levels of job stress in terms of both higher ER ratio (*p* = 2 × 10^−6^) and overcommitment (*p* = 2 × 10^−6^). Due to the study design, physicians with burnout also reported higher levels of burnout symptoms (*p*-values ≤ 7 × 10^−5^) and depressive symptoms (*p* = 5 × 10^−10^) than those without burnout.

**Table 2:** Characteristics of the 60 study participants

Variable	Burnout group (*n* = 30)	Control group (*n* = 30)	*P*-value
Age, years	46.77 (10.56)	52.93 (7.48)	0.012
Body mass index, kg/m^2^	25.63 (3.09)	24.35 (2.72)	0.094
Cardiovascular disease risk score, %	3.11 (1.89)	3.47 (1.89)	0.464
Alcohol, standard drinks/week	3.72 (3.22)	2.93 (2.30)	0.280
Physical activity, times/week	1.99 (1.62)	2.67 (1.92)	0.147
Emotional exhaustion, score	29.17 (7.13)	6.67 (3.99)	2 × 10^−19^
Depersonalization, score	11.33 (7.00)	3.07 (3.60)	8 × 10^−7^
Personal accomplishments, score	12.03 (6.74)	5.67 (4.37)	7 × 10^−5^
Effort-reward ratio	1.34 (0.41)	0.87 (0.27)	2 × 10^−6^
Effort, score	10.65 (1.36)	8.07 (2.13)	6 × 10^−7^
Reward, score	19.58 (4.03)	22.24 (2.96)	0.005
Overcommitment, score	16.65 (2.83)	12.43 (3.37)	2 × 10^−6^
Depressive symptoms, score	7.40 (3.13)	2.20 (1.97)	5 × 10^−10^
MBF, rest, mL/g/min	0.64 (0.13)	0.66 (0.10)	0.530
Coronary flow reserve (CPT)	1.50 (0.52)	1.48 (0.5)	0.838
MBF response (CPT), mL/g/min	0.32 (0.37)	0.33 (0.31)	0.966
Hyperemic MBF (CPT), mL/g/min	0.94 (0.44)	1.00 (0.32)	0.581
Coronary flow reserve (adenosine)	4.31 (1.43)	4.58 (1.09)	0.409
MBF response (adenosine), mL/g/min	2.05 (0.62)	2.27 (0.63)	0.176
Hyperemic MBF (adenosine), mL/g/min	2.66 (0.61)	2.96 (0.65)	0.075

Values given are mean and standard deviation (in parentheses). Normalized values are given for measures of coronary microvascular function. Group differences were calculated with independent samples *t*-test

*CPT* cold pressor test, *MBF* myocardial blood flow

### Myocardial blood flow at rest

Table 3 shows zero-order correlations with MBF at rest. Both higher CVD risk (*r* = 0.270, *p* = 0.037) and higher reward (*r* = 0.314, *p* = 0.014) were significantly associated with higher MBF at rest, whereas higher job stress in terms of a higher ER ratio was significantly associated with lower MBF at rest (*r* =  − 0.335, *p* = 0.009). In regression analysis with age, BMI, burnout, and ER ratio in the model, ER ratio showed an independent association with MBF at rest (unstandardized coefficient *B* =  − 0.108, 95% confidence interval (CI) =  − 0.215, − 0.001; *p* = 0.048); this was not observed for burnout (*p* = 0.18), age (*p* = 0.27), and BMI (*p* = 0.27). Neither burnout (*p* = 0.74) nor OC (*p* = 0.89) was associated with MBF at rest in regression analysis with age (*p* = 0.084), BMI (*p* = 0.15), burnout, and OC as independent variables.

**Table 3:** Zero-order correlations between variables of interest (*n* = 60)

		Cold pressure test			Adenosine challenge		
	MBF at rest	Coronary flow reserve	ΔMBF	Hyperemic MBF	Coronary flow reserve	ΔMBF	Hyperemic MBF
Age	.233	.135	.198	.317*	− .043	.126	.207
Body mass index	− .170	− .336**	− .362**	− .343**	− .212	− .266*	− .279*
CVD risk score	.270*	.109	.165	.275*	− .139	.037	.121
Physical activity	− .032	− .026	− .035	− .023	.142	.121	.118
Alcohol (standard drinks)	− .127	− .183	− .174	− .245	− .002	− .089	− .136
Burnout (yes/no)	− .083	.027	− .006	− .073	− .109	− .177	− .232
Emotional exhaustion	− .097	.001	− .036	− .100	− .154	− .201	− .250
Depersonalization	− .112	.044	.033	− .058	− .003	− .095	− .139
Low personal accomplishments	− .066	− .012	− .031	− .120	− .058	− .122	− .154
Effort-reward ratio	− .335**	− .270*	− .322*	− .408**	− .111	− .323*	− .406**
Effort	− .178	− .292*	− .341**	− .383**	− .162	− .351**	− .411**
Reward	.314*	.113	.145	.225	.021	.153	.223
Overcommitment	− .101	− .261*	− .270*	− .298*	− .133	− .270*	− .299*
Depressive symptoms	− .154	− .223	− .254*	− .290*	− .159	− .266*	− .325*

Values given are Pearson correlation coefficients

*MBF* myocardial blood flow

Significance level: ** *p* < .010, * p < .05

### Endothelium-dependent coronary microvascular function

There were several significant associations of burnout and job stress with CFR, ΔMBF, and hyperemic MBF in the CPT.

#### Zero-order correlations

As can be seen in Table 3, there were significant inverse correlations between job stress and measures of microvascular function of mostly medium effect size (*r* between 0.27 and 0.41). A higher ER-ratio was significantly correlated with lower CRF (*r* =  − 0.270, *p* = 0.037), lower ΔMBF (*r* =  − 0.322, *p* = 0.012), and lower hyperemic MBF (*r* =  − 0.408, *p* = 0.001). Likewise, higher OC was significantly correlated with lower CRF (*r* =  − 0.261, *p* = 0.044), lower ΔMBF (*r* =  − 0.270, *p* = 0.037), and lower hyperemic MBF (*r* =  − 0.298, *p* = 0.021). Of the two ERI components, higher efforts showed a significant association with lower microvascular function for all three measures (*p*-values < 0.023), but reward did not (*p*-values > 0.083). Burnout (yes/no) showed no significant correlation with any measure of microvascular function (*p*-values > 0.58).

#### Independent associations

**Fig. 1:**
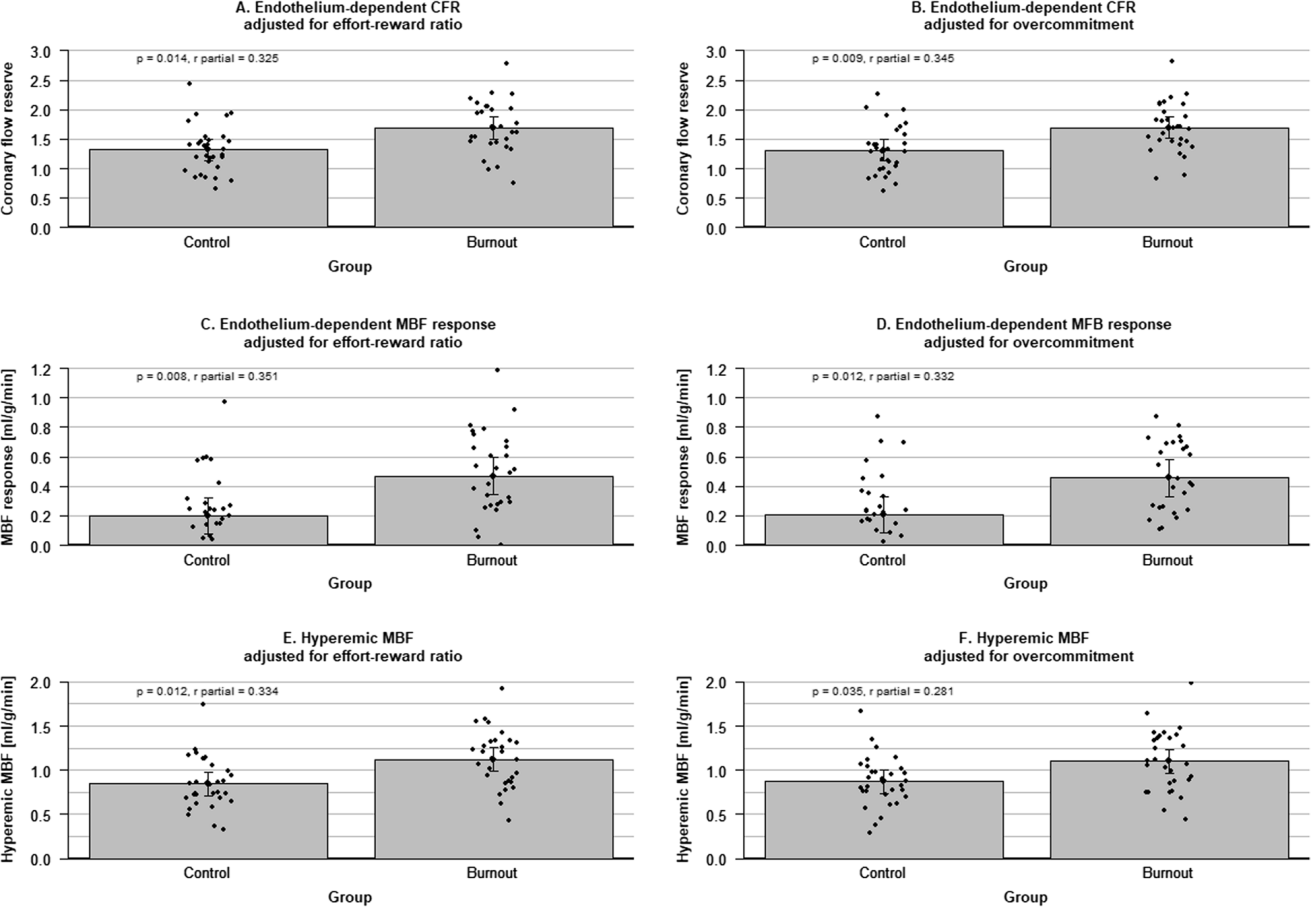
The individual data points and mean values (95% confidence interval) of the primary outcome coronary flow reserve (CFR) and the secondary outcomes myocardial blood flow (MBF) response and hyperemic MBF in response to the cold pressure test between burnout and control physicians. All analyses were adjusted for age and body mass index and either the effort-reward ratio (**A**, **C**, and **E**) or overcommitment scores (**B**, **D**, and **F**). The figure was generated using R version 4.2.0

As shown in Table 4 and Fig. 1, burnout (yes/no) was significantly and independently associated with higher CRF (*r* partial = 0.325, *p* = 0.014), higher ΔMBF (*r* partial = 0.351, *p* = 0.008), and higher hyperemic MBF (*r* partial = 0.334, *p* = 0.012), controlling for age, BMI, and the ER ratio. Likewise, burnout was significantly and independently associated with higher CRF (*r* partial = 0.345, *p* = 0.009), higher ΔMBF (*r* partial = 0.332, *p* = 0.012), and higher hyperemic MBF (*r* partial = 0.281, *p* = 0.035), controlling for age, BMI, and OC.

**Table 4:** Associations of burnout and job stress with endothelium-dependent microvascular function (cold pressor test)

	Coronary flow reserve		ΔMBF		Hyperemic MBF	
	Model for effort-reward ratio	Model for overcommitment	Model for effort-reward ratio	Model for overcommitment	Model for effort-reward ratio	Model for overcommitment
Constant	3.235*** (1.898, 4.572)	3.792*** (2.203, 4.821)	1.597*** (.696, 2.498)	1.657*** (.752, 2.562)	2.067*** (1.096, 3.038)	2.678*** (1.088, 3.086)
Age	.005 (− .008, .019)	.006 (− .007, .018)	.006 (− .004, .015)	.006 (− .002, .015)	.010* (< .001, .020)	.012* (.002, .022)
Body mass index	− .065** (− .108, − .021)	− .068*** (− .107, − .029)	− .049** (− .078, − .020)	− .051*** (− .077, − .024)	− .049** (− .081, − .018)	− .053*** (− .083, − .024)
Burnout (yes/no)	.369* (.076, .661)	.379** (.100, .657)	.270** (.073, .467)	.251* (.058, .443)	.276* (.064, .488)	.230* (.017, .443)
Effort-reward ratio	− .538* (− .969, − .107)		− .421** (− .712, − .131)		− .473** (− .786, − .160)	
Overcommitment		− .055** (− .093, − .017)		− .036** (− .062, − .010)		− .034* (− .063, − .005)
Model statistics	*R*^2^ = .233 *F*(4,54) = 4.109 *P* = .006	*R*^2^ = .276 *F*(4,55) = 5.241 *P* < .001	*R*^2^ = .294 *F*(4,54) = 5.635 *P* < .001	*R*^2^ = .299 *F*(4,55) = 5.874 *P* < .001	*R*^2^ = .329 *F*(4,54) = 6.619 *P* < .001	*R*^2^ = .316 *F*(4,55) = 6.355 *P* < .001

Columns show two multivariable models for each measure of endothelium-dependent microvascular function: one with the effort-reward ratio and the other with the overcommitment scale. Note that the effort-reward ratio and the overcommitment scale were not mutually adjusted, which explains the empty cells in their respective columns. Values given are unstandardized beta-coefficients with 95% confidence interval

*MBF* myocardial blood flow

Significance level: *** *p* < .001, ** *p* < .010, * *p* < .050

**Fig. 2:**
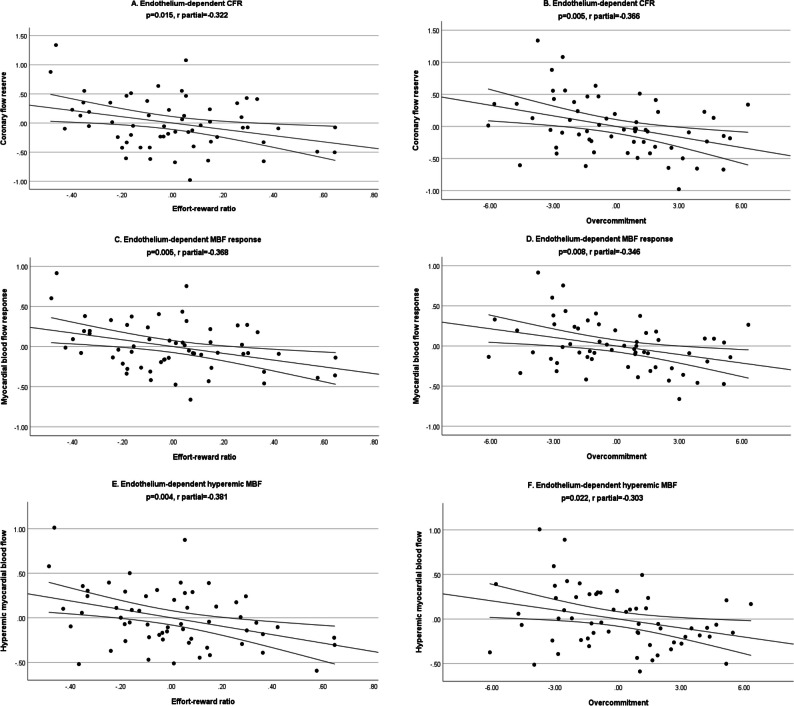
Partial regression plots with fit line (95% confidence interval) for the associations between the effort-reward ratio (**A**, **C,** and **E**) and overcommitment scores (**B**, **D**, and **F**) with the primary outcome coronary flow reserve (CFR) and the secondary outcomes myocardial blood flow (MBF) response and hyperemic MBF in response to the cold pressure test among all participants. All analyses were adjusted for age, body mass index, and burnout (yes/no)

In contrast, as shown in Table 4 and Fig. 2, higher job stress, measured by the ER ratio, was significantly and independently associated with lower CRF (*r* partial =  − 0.322, *p* = 0.015), lower ΔMBF (*r* partial =  − 0.368, *p* = 0.005), and lower hyperemic MBF (*r* partial =  − 0.381, *p* = 0.004). Job stress measured by the OC score was also significantly and independently associated with lower CRF (*r* partial =  − 0.366, *p* = 0.005), lower ΔMBF (*r* partial =  − 0.346, *p* = 0.008), and lower hyperemic MBF (*r* partial =  − 0.303, *p* = 0.022). The majority of adjusted correlation coefficients exceeded 0.3, with these independent associations indicating a medium effect size.

Follow-up analyses with individual burnout dimension revealed that higher EE was significantly and independently associated with greater microvascular function regarding all three measures (*r* partial between 0.280 and 0.329, p-values between 0.013 and 0.037). These associations held true after adjusting for age, BMI, and either the ER ratio (Table 5, Model 1) or OC score (Table 5, Model 2). Similar significant and independent associations with greater microvascular function were observed for higher DP (*r* partial between 0.261 and 0.317, *p*-values between 0.016 and 0.049), except for the association between DP and CFR (*p* = 0.051) (Table 5, Model 1); PA showed no significant association with microvascular function measures (*p*-values > 0.09). Of the ERI components, higher efforts showed significant and independent associations with lower microvascular function with all three measures (Table 5, Models 3 and 4: *r* partial between − 0.414 and − 0.372, *p*-values between 0.001 and 0.005), but reward did not (*p*-values > 0.32).

**Table 5:** Associations of burnout and job stress dimensions with endothelium-dependent microvascular function (cold pressor test)

	Coronary flow reserve	ΔMBF	Hyperemic MBF
Emotional exhaustion			
Model 1	.013 (.001, .024)*	.009 (.001, .017)*	.010 (.001, .018)*
Model 2	.015 (.003, .027)*	.010 (.001, .018)*	.010 (.001, .018)*
Depersonalization			
Model 1	.020 (< − .001, .040)	.016 (.003, .029)*	.015 (< .001, .029)*
Model 2	.022 (.002, .041)*	.016 (.003, .030)*	.015 (< .001, .029)*
Effort			
Model 3	− .102 (− .168, − .036)**	− .075 (− .120, − .031)**	− .076 (− .126, − .027)**
Model 4	− .100 (− .168, − .033)**	− .074 (− .120, − .029)**	− .073 (− .123, − .023)**

Values given are unstandardized beta-coefficients with 95% confidence interval

Model 1 controlled for age, body mass index (BMI), and effort-reward ratio. Model 2 controlled for age, BMI, and overcommitment. Model 3 controlled for age, BMI, and group (burnout yes/no). Model 4 controlled for age, BMI, group, and reward

*MBF* myocardial blood flow

Significance level: ** *p* < .010, * p < .050

### Endothelium-independent microvascular function

With the adenosine challenge, one participant in the burnout group, but none in the control group, had CFR < 2. Zero-order correlations (Table 3) showed that higher job stress, measured by the ER ratio, was significantly correlated with lower ΔMBF (*r* =  − 0.323, *p* = 0.012) and lower hyperemic MBF (*r* =  − 0.406, *p* = 0.001). Similarly, a higher OC score was also correlated with lower ΔMBF (*r* =  − 0.270, *p* = 0.037) and lower hyperemic MBF (*r* =  − 0.299, *p* = 0.020). Of the ERI components, the effort, but not the reward scale, showed a significant correlation with ΔMBF (*r* =  − 0.351, *p* = 0.006) and hyperemic MBF (*r* =  − 0.411, *p* = 0.001). These correlations were mostly of medium effect size. However, as can be seen in Table 6, none of the significant zero-order correlations maintained significance in multivariable regression models (*p*-values > 0.05). Burnout (yes/no) showed no significant independent association with any measure of endothelium-independent coronary microvascular function (*p*-values > 0.69).

**Table 6:** Associations of burnout and job stress with endothelium-independent microvascular function (adenosine challenge)

	Coronary flow reserve		ΔMBF		Hyperemic MBF	
	Model for effort-reward ratio	Model for overcommitment	Model for effort-reward ratio	Model for overcommitment	Model for effort-reward ratio	Model for overcommitment
Constant	7.207*** (3.260, 11.154)	8.079*** (4.178, 11.979)	3.821*** (1.965, 5.676)	4.071*** (2.217, 5.925)	4.332*** (2.499, 6.166)	4.513*** (2.650, 6.377)
Age	− .010 (− .050, .029)	− .013 (− .052, .025)	.002 (− .017, .021)	.003 (− .015, .021)	.006 (− .012, .024)	.008 (− .010, .027)
Body mass index	− .078 (− .206, .050)	− .086 (− .202, .030)	− .050 (− .110, .010)	− .057* (− .113, .002)	− .049 (− .108, .010)	− .061* (− .116, − .006)
Burnout (yes/no)	− .141 (− 1.003, .722)	− .008 (− .838, .822)	.081 (− .324, .487)	.063 (− .331, .458)	.067 (− .334, .467)	.010 (− .387, .406)
Effort-reward ratio	− .210 (− 1.483, 1.063)		− .511 (− 1.109, .087)		− .578 (− 1.169, .014)	
Overcommitment		− .056 (− .168, .056)		− .045 (− .099, .008)		− .042 (− .095, .012)
Model statistics	*R*^2^ = .044 *F*(4,54) = .624 *P* = .647	*R*^2^ = .069 *F*(4,55) = 1.024 *P* = .403	*R*^2^ = .119 *F*(4,54) = 1.824 *P* = .138	*R*^2^ = .142 *F*(4,55) = 2.272 *P* = .073	*R*^2^ = .154 *F*(4,54) = 2.465 *P* = .056	*R*^2^ = .175 *F*(4,55) = 2.916 *P* = .029

Columns show two multivariable models for each measure of endothelium-independent microvascular function: one with the effort-reward ratio and the other with the overcommitment scale. Note that the effort-reward ratio and the overcommitment scale were not mutually adjusted, which explains the empty cells in their respective columns. Values given are unstandardized beta-coefficients with 95% confidence interval

*MBF* myocardial blood flow

Significance level: *** *p* < .001, * *p* < .050

### Complementary analyses

The interaction between the ER ratio and OC showed no independent association with any measure of microvascular function (*p*-values > 0.16). Likewise, the interaction between burnout (yes/no) and the ER ratio and the interaction between burnout (yes/no) and OC were not significant for any measure of microvascular function (*p*-values > 0.20).

## Discussion

We found that both burnout and job stress showed significant and independent, albeit opposing, associations with endothelium-dependent coronary microvascular function in male physicians. Specifically, burnout exhibited positive associations with endothelium-dependent CFR, the primary outcome variable in our study, MBF response, and hyperemic MBF. In turn, job stress, defined by the ER ratio and OC, showed negative associations with endothelium-dependent CFR, MBF response, and hyperemic MBF. The magnitude of the observed effects suggests that these associations may be of clinical relevance. When examining the individual dimensions of burnout and job stress, EE, DP, and efforts were significantly and independently associated with coronary microvascular endothelial function. To put it simply, coronary microvascular endothelial function was better in male physicians versus those without burnout, but reduced in those with higher job stress.

To our knowledge, endothelial function has not previously been investigated in burnout, whereas, consistent with our findings, paramedics with high job strain were shown to have endothelial dysfunction measured by peripheral artery tonometry [34]. Our finding of and association of endothelial-dependent coronary microvascular dysfunction with higher ER ratio or OC aligns with meta-analyses showing a link between various aspects of job stress, including ERI, and an increased CHD risk [10, 14, 35–37]. While the ER ratio has been extensively studied in relation to CHD risk, OC has received comparatively less attention [12, 38]. However, OC has been associated with subclinical atherosclerosis in Chinese workers [39] and an increased CHD risk in patients referred for coronary angiography [40] and restenosis in men following coronary angioplasty [41]. We therefore interpret that job stress resulting both extrinsically from the work environment and intrinsically from an exhaustive pattern of coping with demanding work-related situations could be associated with poor cardiovascular health in male physicians. High effort was significantly associated with microvascular endothelial function whereas low reward was not. In contrast, we found no evidence for the theoretical concept [30] that the association between higher ER ratio and lower microvascular endothelial function would be strongest in male physicians scoring high on OC. This concurs with the majority of empirical investigations in which OC was not found to be a moderator of the association between ERI and various health outcomes [42].

Regarding clinical implications, effective psychosocial interventions to manage job stress in physicians [43] could be tested for a potential benefit on microvascular endothelial dysfunction. Such research may be informed by a previous trial showing that 12 weeks of meditation practice improved PET-assessed CFR in CHD patients [44]. Another study in patients with stable CHD showed that the combination of usual care and weekly 1.5-h stress management sessions for 16 weeks resulted in greater improvement in endothelial function (i.e., flow-mediated dilation) than usual care alone [45].

As burnout has been associated with an increased risk of CHD [11], the observation that burnout was related to increased endothelium-dependent coronary microvascular function seems counterintuitive. Although speculative, we offer some biobehavioral explanations for interpreting the finding of apparently improved endothelium-dependent coronary microvascular function in the burnout group. Based on a previous study in which male patients with burnout exhibited higher systolic blood pressure during psychosocial stress than healthy male controls [46], there could be an increased sympathetic reaction to the CPT in our male physicians with burnout. This hypothesis gains support from the observation that resting MBF was not different between physicians with burnout and those without, yet significantly lower in physicians with a higher ER ratio. At least during the acute phase, the burnout syndrome could possibly have a protective effect on cardiovascular health against high levels of stress via inhibiting even higher levels of stress and effort. Specifically, sympathetic activity has been associated with increased low-grade inflammation [47] and there is a sequence leading first to endothelial proinflammatory activation, which precedes endothelial dysfunction in atherogenesis [48]. Therefore, systemic inflammation in burnout [15] due to sympathetic activity [46] might be associated with endothelial activation rather than dysfunction in our participants. Longitudinal studies could investigate whether the positive relationship between burnout and coronary microvascular endothelial function changes to a negative one with a longer duration of burnout symptomatology. This relationship could be further explored, particularly with a focus on investigating the role of the sympathetic nervous system and low-grade inflammation. This may particularly be the case for individuals with high EE and high DP, both of which were associated with microvascular function in our study and an increased CVD risk in a large population from Finland [49].

In statistical terms, the observation that the effect of burnout on coronary microvascular endothelial function only emerged in the fully adjusted model suggests a suppressor effect. The inclusion of job stress likely improved the model’s predictive power by accounting for the confounding relationship between burnout and job stress, thereby revealing a “true” association between burnout and microvascular function. We found no evidence that burnout modified the association between job stress and microvascular endothelial function. By all means, examining the independent associations of burnout and job stress in the same study could provide a more comprehensive understanding of the complex relationships between work-related stressors and workers’ cardiovascular health. It should be noted, however, that CFR and hyperemic MBF and MBF response without adjustment for covariates did not show significant group differences. This could be due to the small sample size or to the fact that burnout does not really affect coronary microvascular function.

We found no significant independent association of burnout and job stress with endothelial-independent coronary microvascular function as assessed through an adenosine challenge. The selective impairment of endothelium-dependent regulation of CFR, MBF response, and hyperemic MBF indicates a specific dysfunction in the endothelium’s ability to respond to sympathetic stimuli (e.g., the CPT used in our study), and release vasodilators, like nitric oxide. The endothelium plays a crucial role in the mental stress-induced coronary microvascular response [50], and impaired endothelium-dependent coronary vasodilation is linked to exercise-induced myocardial ischemia [51]. Therefore, in individuals with high job stress, there could be increased vulnerability to acute coronary events when there is a temporary rise in sympathetic activity due to, for instance, acute emotional stress or intense physical exertion [52–54].

While we made efforts to match physicians with burnout to controls by age, it is worth noting that the burnout group turned out to be younger, consistent with findings from large population-based studies showing an inverse association between burnout and age [55, 56]. This age difference may be partly explained by the “healthy worker effect” [55, 56], a phenomenon observed when employees with burnout leave their jobs or professions at a younger age than those who remain in the workforce without health issues. It is possible that older physicians have developed better strategies for coping with job-related stress, or there may be differences in training among different cohorts of physicians.

The use of PET to quantify microvascular function with established techniques and validated questionnaires to limit measurement bias, and the inclusion of a well-defined group of male physicians with and without clinical burnout were strengths of our study. However, this study also has several noteworthy limitations. The calculation of the target sample size was not based on power analysis for the primary study outcome. The criteria for matching between the burnout and control groups, along with the exclusion criteria related to numerous health conditions, potentially affecting both cardiovascular health and workplace-related variables, aimed to minimize residual confounding. However, the sample size allowed only a limited number of covariates to be included in the multivariable analyses, so residual confounding cannot be excluded, resulting in some level of uncertainty in our findings. For instance, the inclusion of inflammatory biomarkers could have provided additional valuable information. Therefore, the study was likely underpowered and the results should be considered hypothesis-generating. Moreover, while we made adjustment for multiple comparisons for the primary outcome CFR, the additional examination of multiple exposures and multiple outcomes in our study was exploratory and raises the possibility that some *p*-values became significant by chance alone, so these results should be interpreted with caution. The study employed a cross-sectional design, aimed to provide a snapshot of current relationships between work-related variables and microvascular function, facilitating the identification of patterns of association. However, the deliberate choice of this design limits the possibility of inferring causality or mechanisms and ruling out reverse causality. Existing literature and conceptual considerations substantiate the idea that job stress and burnout are risk factors for CVD development. Notably, our study participants were free of CVD and reported experiencing undue exhaustion and job stress for a minimum of 6 months before the study. While these inclusion criteria may alleviate concerns about reverse causality, it does not preclude the possibility that subclinical CVD contributed to exhaustion, requiring increased efforts to maintain job performance. To enhance the robustness of our findings, we emphasize the need for future longitudinal studies to examine the temporal relationships between burnout, job stress, and coronary microvascular function. The specificity of the study population increased the study’s internal validity, but the numerous exclusion criteria limited the sample size affecting the generalizability of the results. We only included male physicians without a history of CVD, so it is unclear whether the findings generalize to other occupational groups, patients with established CVD, and particularly female physicians. This is important because, in response to acute stress, pre-menopausal women show both smaller autonomic responses [57] and greater endothelial dysfunction [58] than men of the same age.

## Conclusions

The present study provides some evidence that burnout and job stress show opposite associations with coronary microvascular endothelial function in male physicians. Longitudinal studies with larger sample sizes are preferably needed to determine the transferability of our findings to other populations, including female physicians, and their clinical relevance for targeted interventions to reduce CHD risk in the working population.

## Data Availability

The datasets used and analyzed during the current study are available from the corresponding author on reasonable request.

## Abbreviations

BMI: Body mass index
CFR: Coronary flow reserve
CHD: Coronary heart disease
CPT: Cold pressor test
CVD: Cardiovascular disease
DP: Depersonalization
EE: Emotional exhaustion
ERI: Effort-reward imbalance
MBF: Myocardial blood flow
MBI-HSS: Maslach Burnout Inventory-Human Services Survey
OC: Overcommitment
PA: Personal accomplishment
PET-CT: Positron emission tomography computed tomography
PHQ: Patient Health Questionnaire
SCORE: Systematic Coronary Risk Evaluation
VIF: Variance inflation factor
