# Correction: Coronary microvascular function in male physicians with burnout and job stress: an observational study

**DOI:** 10.1186/s12916-024-03290-6

**Published:** 2024-02-16

**Authors:** Roland von Känel, Mary Princip, Sarah A. Holzgang, Chrysoula Garefa, Alexia Rossi, Dominik C. Benz, Andreas A. Giannopoulos, Philipp A. Kaufmann, Ronny R. Buechel, Claudia Zuccarella-Hackl, Aju P. Pazhenkottil

**Affiliations:** 1 Department of Consultation-Liaison Psychiatry and Psychosomatic Medicine, University Hospital Zurich, University of Zurich, Culmannstrasse 8, CH-8091 Zurich, Switzerland; 2 Cardiac Imaging, Department of Nuclear Medicine, University Hospital Zurich, University of Zurich, Zurich, Switzerland; 3 Department of Cardiology, University Hospital Zurich, University of Zurich, Zurich, Switzerland

**Correction** **:** **BMC Med 21, 477 (2023)**

**https://doi.org/10.1186/s12916-023-03192-z**

The original article [1] contains errors in the following sentence in the ‘Burnout’ sub-section of the ‘Methods’ section:

“Participants rated each item on a scale ranging from 1 (“never”) to 7 (“daily”)”.

Numbers ‘1’ and ‘7’ in the sentence should instead respectively state '0' and '6'.
